# Human recombinant follicle stimulating hormone (rFSH) compared to urinary human menopausal gonadotropin (HMG) for ovarian stimulation in assisted reproduction: a literature review and cost evaluation

**DOI:** 10.1007/s40618-014-0204-4

**Published:** 2014-12-06

**Authors:** P. E. Levi Setti, C. Alviggi, G. L. Colombo, C. Pisanelli, C. Ripellino, S. Longobardi, P. L. Canonico, G. De Placido

**Affiliations:** 1 Humanitas Fertility Center, Division of Gynaecology and Reproductive Medicine, Department of Gynaecology, Humanitas Research Hospital, Rozzano, Milan Italy; 2 Dipartimento Universitario di Neuroscienze, Scienze Riproduttive ed Odontostomatologiche, Università degli Studi di Napoli, “Federico II”, Naples, Italy; 3 Department of Drug Sciences, University of Pavia, Pavia, Italy; 4 S.A.V.E. Studi Analisi Valutazioni Economiche, Milan, Italy; 5 Hospital Pharmacist, ACO San Filippo Neri, Rome, Italy; 6 Società Italiana Di Farmacia Ospedaliera, Milan, Italy; 7 CSD Medical Research Srl, Viale Jenner n 53, 20159 Milan, Italy; 8 Medical Department, MerckSerono S.p.A, Rome, Italy; 9 Dipartimento di Scienze del Farmaco, Università del Piemonte Orientale, Largo Donegani 2, Novara, Italy; 10 University Department of Obstetrics, Gynaecology, Urology and Reproductive Medicine, University of Naples Federico II, Naples, Italy

**Keywords:** FSH, HMG, Human menopausal gonadotropin, Recombinant follicle stimulating hormone, Systematic review, Cost evaluation, Gonadotropins, Infertility, Assisted reproduction

## Abstract

**Background:**

Gonadotropins are protein hormones which are central to the complex endocrine system that regulates normal growth, sexual development, and reproductive function. There is still a lively debate on which type of gonadotropin medication should be used, either human menopausal gonadotropin or recombinant follicle-stimulating hormone. The objective of the study was to perform a systematic review of the recent literature to compare recombinant follicle-stimulating hormone to human menopausal gonadotropin with the aim to assess any differences in terms of efficacy and to provide a cost evaluation based on findings of this systematic review.

**Methods:**

The review was conducted selecting prospective, randomized, controlled trials comparing the two gonadotropin medications from a literature search of several databases. The outcome measure used to evaluate efficacy was the number of oocytes retrieved per cycle. In addition, a cost evaluation was performed based on retrieved efficacy data.

**Results:**

The number of oocytes retrieved appeared to be higher for human menopausal gonadotropin in only 2 studies while 10 out of 13 studies showed a higher mean number of oocytes retrieved per cycle for recombinant follicle-stimulating hormone. The results of the cost evaluation provided a similar cost per oocyte for both hormones.

**Conclusions:**

Recombinant follicle-stimulating hormone treatment resulted in a higher oocytes yield per cycle than human menopausal gonadotropin at similar cost per oocyte.

## Introduction

Gonadotropins are protein hormones secreted by gonadotrope cells of the anterior pituitary of vertebrates [1], which are central to the complex endocrine system that regulates normal growth, sexual development, and reproductive function. The two key hormones, follicle-stimulating hormone (FSH) and luteinizing hormone (LH), act synergistically in reproduction, stimulating the growth and recruitment of immature ovarian follicles in the ovary and primary spermatocytes in the testis to undergo the first division of meiosis and to form secondary spermatocytes, in women and men, respectively.

Gonadotropin treatments can be used to stimulate ovulation in women with low natural gonadotropin or estrogen levels, when clomiphene treatment has been ineffective in regulating ovulation caused by polycystic ovary syndrome, for developing multiple egg follicles in the ovaries (retrieved and used in assisted reproductive techniques), in combination with intrauterine insemination for couples with unexplained infertility when clomiphene was not effective. In men, gonadotropin therapy can improve low sperm counts caused by low levels of natural gonadotropins.

For this reason, gonadotropin medications have been the cornerstone of infertility treatment since 1950, when human menopausal gonadotropin (HMG) was first introduced into clinical practice [2], but clinical trials started only in the 1960s [3, 4] . A first alternative medication to HMG, which contained an equal ratio of FSH and LH, became available in the late 1960s; following different purification processes, urinary FSH (uFSH) was still urine‐derived, but largely purified of LH [5]. The final product contained 150 IU of FSH and 1 IU of LH per milligram of protein (though not of co‐purified proteins). Further technological advances made it possible to obtain uFSH with even less amount of LH, and in the 1990s highly purified FSH (HP-FSH), which contains <0.1 IU of LH activity and <5 % of unidentified urinary proteins, and highly purified HMG (HP-HMG), with the same labeled ratio of FSH: LH activity of HMG, became available [6, 7].

In the late 1990's, a different type of gonadotropin had been developed: using recombinant DNA technology, recombinant FSH (rFSH) was produced, obtaining preparations that have high purity and biological potency and are completely LH free [8, 9].

Following commercialization of recombinant FSH, there has been much controversy with regard to the type of gonadotropin which should be utilized. The present manuscript presents a systematic review of the literature comparing rFSH and HMG with the aim of determining differences in efficacy between these two compounds, as well as, a cost evaluation conducted from the findings of the review.

## Materials and methods

The analysis in this article is based on previously conducted studies, and does not involve any new studies of human or animal subjects performed by any of the authors.

### Identification of literature

To assess the efficacy of urinary HMG and rFSH therapies, a literature search of the National Library of Medicine and the National Institutes of Health (PubMed), Medline and Cochrane Controlled Trials Register (i.e., CENTRAL, The Cochrane Library) electronic databases was performed using the following keywords: ‘HMG’, ‘human menopausal gonadotropin’, ‘recombinant follicle stimulating hormone’ and ‘recombinant FSH’. Only prospective, randomized, controlled trials comparing recombinant FSH versus HMG treatments with an adequate sample size were included, assuming a population of at least 15 women by arms to avoid potential bias due to considering small studies. No additional selection on patients characteristics, indications, treatment protocols were applied. Studies selected for inclusion in the review were identified by two experienced health economists (C. Ripellino and A. Guasconi); the reference lists of review articles and included studies drafted by each researcher were compared in order to set the final eligible studies list. No attempts were made to contact authors for additional information.

### Study selection and outcome

The outcome measure used to evaluate treatment efficacy was the number of oocytes retrieved per cycle.

This outcome was chosen since the number of oocytes retrieved is directly associated with the stimulating effect of gonadotropins, while other outcomes, such as live birth rate, depend not only on gonadotropins but also on many other interventions and factors (e.g., male factor, quality of laboratory), making it difficult to create a direct cause–effect relation between ovarian stimulation and live birth rate.

Moreover, some studies investigated the association between egg number and live birth rate following in vitro fertilization treatments and suggested that the number of eggs is a robust surrogate parameter for clinical success [10, 11].

**Fig. 1:**
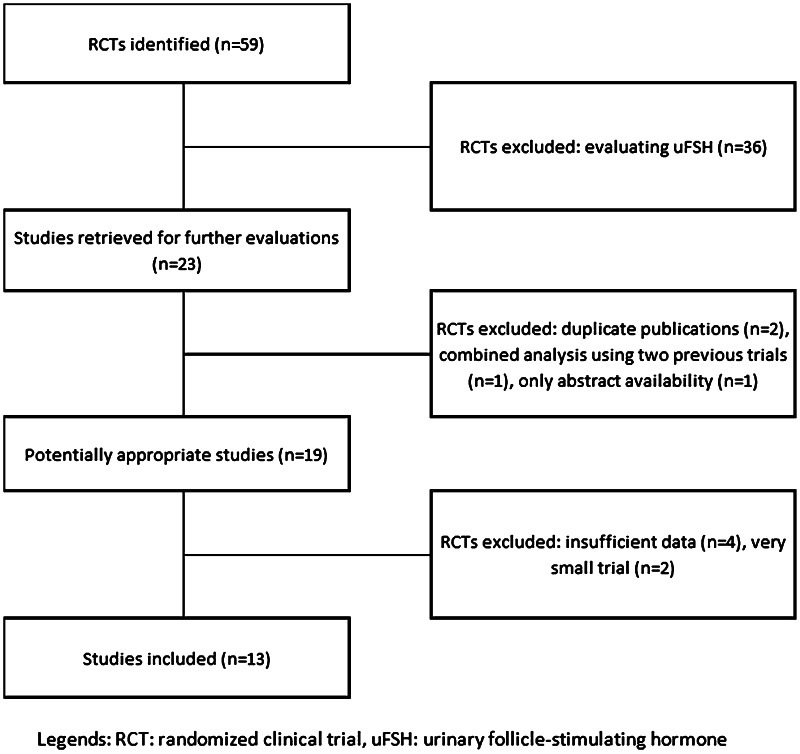
Identification and selection of the studies to be included

A total of 59 articles were found (Fig. 1). Subsequently, 46 articles were excluded for the following reasons: urinary FSH versus recombinant FSH treatment (*n* = 36), duplicate publications (*n* = 2), combined analysis using two previous trials (*n* = 1), abstract availability only (*n* = 1), no oocytes outcome (*n* = 4), very small trial (less than 30 patients, *n* = 2). The remaining 13 studies were considered for this publication [12–24] (Table 1).

**Table 1:** Characteristics of included studies

	Population	Interventions
Jansen et al. [12]	109 Women undergoing IVF	rFSH vs HMG at a starting dose of 150 IU for rFSH and 225 IU for HMG
Gordon et al. [13]	128 Women undergoing IVF	rFSH vs HMG at a starting dose of 225 IU in a long luteal GnRHa protocol
NG et al. [14]	40 Women undergoing ICSI	rFSH vs HMG at a starting dose of 300 IU for 2 days, then 150 IU
Strehler et al. [15]	578 Women undergoing IVF or ICSI	rFSH vs HMG at a starting dose from 150 to 450 IU
Westergaard et al. [16]	379 Women undergoing IVF	rFSH vs HMG at a starting dose of 225 IU in a long luteal GnRHa protocol
Balash et al. [17]	60 Patients undergoing ICSI and having unexplained or male-related primary infertility	rFSH vs HMG at a starting dose of 150 IU in a long luteal GnRHa protocol
Kilani et al. [18]	100 Women undergoing IVF	rFSH vs hp-HMG at a starting dose of 150 IU in a GnRHa protocol
Rashidi et al. [19]	60 Women undergoing ICSI	rFSH vs HMG at a starting dose of 150 IU
Andersen et al. [20]	731 Infertile women undergoing IVF	rFSH vs hp-HMG at a starting dose of 225 IU in a GnRH-antagonist protocol
Bosch et al. [21]	280 Infertile women undergoing IVF or ICSI	rFSH vs hp-HMG at a starting dose of 225 IU in a fixed GnRH-antagonist protocol
Hompes et al. [22]	629 Infertile women undergoing IVF	rFSH vs hp-HMG at a starting dose of 150 IU in a GnRH-a long protocol
Devroey et al. [23]	749 Infertile patients undergoing ICSI	rFSH vs hp-HMG at a starting dose of 150 IU in a GnRH-antagonist protocol
Ye et al. [24]	127 Infertile women undergoing IVF or ICSI	rFSH vs hp-HMG at a starting dose of 225 IU

*IVF* in vitro fertilization, *ICSI* intracytoplasmic sperm injection, *rFSH* recombinant follicle stimulating hormone, *hp-HMG* highly purified human menopausal gonadotropin, *IU* international units, *GnRH* gonadotropin-releasing hormone, *GnRH-a* gonadotropin-releasing hormone agonist

### Cost analysis

Cost calculations were performed using Italian treatment costs and findings from literature review. Starting from individual studies total dose (IU) and number of oocytes retrieved per cycle, then applying Italian gonadotropin prices, it was possible to obtain the cost per oocyte for each study.

Unit costs used were obtained from the price database available on the Codifa Database [25], last updated in January 2014, i.e. €38.58 per vial of rFSH, and €26.57 per vial of HMG. Only gonadotropin costs were considered, assuming the cost of other resources to be identical or captured by treatment charges that do not differentiate between stimulation protocols.

## Results

### Efficacy

About half of the included studies found that rFSH was associated with a lower mean total dose in comparison with HMG (Westergaard et al. [16], Rashidi et al. [19], Andersen et al. [20], Hompes et al. [22], Devroey et al. [23] and Ye et al. [24]), while in other publications (Jansen et al. [12], Gordon et al. [13], NG et al. [14], Strehler et al. [15], Balash et al. [17], Kilani et al. [18] and Bosch et al. [21]) HMG was associated with a lower total mean dose (Table 2).

**Table 2:** Table of outcome measures

	Total dose (IU)		No. of retrieved oocytes	
	rFSH (means ± std)	HMG (means ± std)	rFSH (means ± std)	HMG (means ± std)
Jansen et al. [12]	1,410 ± 228	1,365 ± 228	11.2 ± 6.8	8.3 ± 6.2
Gordon et al. [13]	2,025 ± 350	1,981 ± 570	12 ± 6	10 ± 7
NG et al. [14]	1,800 ± 270	1,650 ± 270	12.6 ± 8.9	9.6 ± 8.1
Strehler et al. [15]	2,150 ± 797	1,516 ± 545	12.29 ± 7.8	9.67 ± 5.92
Westergaard et al. [16]	2,242 ± 375	2,280 ± 435	12.9 ± 6.8	12.9 ± 6.7
Balash et al. [17]	2,449 ± 885	1,922 ± 379	11.79 ± 4.55	9.1 ± 4.35
Kilani et al. [18]	2,025 ± 795	1,680 ± 530	6.8 ± 3.9	7.9 ± 4.6
Rashidi et al. [19]	2,138 ± 800	2,250 ± 800	8.7 ± 8.5	9 ± 6.2
Andersen et al. [20]	2,385 ± 622	2,508 ± 729	11.8 ± 5.7	10.0 ± 5.4
Bosch et al. [21]	2,624 ± 801	2,481 ± 994	14.4 ± 8.1	11.3 ± 6.0
Hompes et al. [22]	1,759.7	1,821.0	10.56	7.76
Devroey et al. [23]	1,353 ± 296	1,433 ± 371	10.7 ± 5.8	9.1 ± 5.2
Ye et al. [24]	2,162.7 ± 399.4	2,219 ± 502.7	10.2 ± 5.2	7.2 ± 4.2

The mean total dose ranged from 1353 IU to 2624 IU for rFSH and from 1365 IU to 2508 IU for HMG. The main outcome, i.e., the number of oocytes retrieved, was observed to be higher for HMG in 2 studies only (Kilani et al. [18] and Rashidi et al. [19]); 10 out of 13 studies showed a higher mean number of oocytes for rFSH, while Westergaard et al. [16] found the same mean value for both rFSH and HMG. The mean number of oocytes retrieved ranges from 6.8 to 14.4 for rFSH and from 7.2 to 12.9 for HMG (Table 2).

### Costs

Results of the economic evaluation are presented in Table 3. The ovarian stimulation with rFSH compared to HMG generated a cost per oocyte that varies from €65 to €153 for recombinant therapy and from €55 to €109 for urinary therapy; thus, the difference between therapies ranges from −€0.1 to €77.

**Table 3:** Table of costs

	Cost per oocyte		
	rFSH	HMG	Difference in costs
Jansen et al. [12]	€ 64.8	€ 58.3	€ 6.5
Gordon et al. [13]	€ 86.8	€ 70.2	€ 16.6
NG et al. [14]	€ 73.5	€ 60.9	€ 12.6
Strehler et al. [15]	€ 90.0	€ 55.5	€ 34.4
Westergaard et al. [16]	€ 89.4	€ 62.6	€ 26.8
Balash et al. [17]	€ 106.9	€ 74.8	€ 32.0
Kilani et al. [18]	€ 153.2	€ 75.3	€ 77.8
Rashidi et al. [19]	€ 126.4	€ 88.6	€ 37.8
Andersen et al. [20]	€ 104.0	€ 88.9	€ 15.1
Bosch et al. [21]	€ 93.7	€ 77.8	€ 16.0
Hompes et al. [22]	€ 85.7	€ 83.1	€ 2.6
Devroey et al. [23]	€ 65.0	€ 55.8	€ 9.3
Ye et al. [24]	€ 109.1	€ 109.2	−€ 0.1

A relevant difference in the cost per oocyte between rFSH and HMG has been observed in Kilani et al. (€ 77) [18]; this cost difference is greater than the values of the other studies, which varied from −€0.1 to €37.

## Discussion

This review evaluated efficacy in terms of number of oocytes retrieved per cycle and the costs, calculated applying Italian treatment costs to the findings of the retrieved studies, of rFSH and HMG in ovarian stimulation protocols in infertile women. The results of this systematic review suggest that rFSH is likely to be more effective than HMG. The number of oocytes retrieved per cycle was higher in almost all studies considered, with a similar total dose used for both rFSH and HMG.

The results of the economic evaluation provided a similar cost per oocyte for rFSH and HMG (with maximum cost differences of €37 for Rashidi et al. [19] and €77 for Kilani et al. [18]).

The present study findings are in agreement with the results of the largest meta-analyses published to date on this subject [26–29].

The Cochrane review by van Wely et al. [26] comparing rFSH to other gonadotropins irrespective of the downregulation protocol used, presented evidence of a major oocyte production for rFSH in comparison with HMG in most of the considered studies.

In the 5 trials included in the meta-analysis conducted by Jee et al. [27], more oocytes were retrieved in the rFSH group, with the exception of the trial by Kilani et al. [18]. In the meta-analysis by Lehert et al. [28], treatment with human menopausal gonadotropins resulted in fewer oocytes (mean difference −1.54; 95 % CI −2.53 to −0.56; *p* < 0.0001) compared to rFSH. Furthermore, a higher total dose of human menopausal gonadotropin was necessary [mean difference 235.46 IU (95 % CI 16.62–454.30; *p* = 0.03)].

A meta-analysis conducted by Wex et al. [29] showed a greater number of oocytes with rFSH (mean difference 1.96; 95 % CI 1.02–2.90). Furthermore, authors developed a cost-minimization model where rFSH has been found to be cost-saving, at 90,195 kr (€10,282 or $13,394) with rhFSH compared to 96,436 kr (€10,994 or $14,321) with HP-HMG per live-birth.

A retrospective databases chart review from 4 European countries investigated gonadotropins usage, oocyte and embryo yield, and pregnancy outcomes in IVF cycles using rFSH or HP-HMG have been conducted by Trew et al. [30]. The group demonstrated a significantly lower drug usage per cycle for rFSH than HP-HMG (22.6 % higher for HP-HMG; *p* < 0.01) and a significantly greater average oocyte yield per IVF cycle in patients treated with rFSH in comparison with HP-HMG (10.80 ± 6.02 for rFSH vs. 9.77 ± 5.53 for HP-HMG; *p* < 0.01).

The economic evaluation presented in this study shows a similar cost per oocyte for rFSH and HMG, is the first costs analysis performed using Italian treatment costs. The minor cost difference found suggests that the higher unit cost of rFSH may be offset by a higher efficacy compared with HMG. Furthermore, clinicians should bear in mind that rFSH allows for more frozen embryo transfers than HMG, since it produces a greater number of oocytes; thus, it can be possible to reduce the number of ovarian stimulations, with a consequent minor overall treatment cost.

Additionally, Zhu et al. [31] found that embryo cryopreservation and subsequent transfer cycle under optimal conditions, as opposed to fresh transfer cycle, achieve improved synchrony between embryo and endometrial development, thereby enhancing the clinical outcome.

The safety and tolerability of rFSH have been extensively evaluated since it became available. The most obvious clinical safety advantages arise from the high purity of rFSH; rFSH has been proven to have better overall tolerability than any previous FSH preparation [32]. In fact, filled by mass manufacturing process of follitropin *α* eliminates the intrinsic variability of the rat bioassay and ensures high batch-to-batch and vial-to-vial consistency of rFSH content. Furthermore, analytical assessment of commercially available rFSH pharmaceutical products has shown that follitropin *α* filled by mass is the most consistent rFSH in terms of protein content [33].

In contrast, since HMG preparations are directly extracted from human urine, the FSH activity in the preparations is highly variable between batches; the control of raw material of the individual contributors and the variation of purification processes are the major barriers in improving the quality of urinary preparations [34]. Systematic literature reviews provide an excellent method to address eventual deficiencies of individual trials by considering several clinical studies. However, differences in results among studies could exist and could depend on clinical trials with different design and clinical practice, rather than differences in participants and clinical settings.

However, this review presents some limitations. Only peer-reviewed papers are included, hence there is the possibility of selection bias related to the publication source. In addition, even though the searches are done thoroughly through multiple major databases with cross-referencing, there is a possibility that some papers with pivotal findings for this issue have not been included in this current review. However, since no selection criteria on patients’ characteristics, indications, or treatment protocols were applied, publications selected cover a wide range of interventions and medical settings, which are representative of the use of gonadotropins.

Using just the number of oocytes retrieved per cycle as the only outcome to evaluate the efficacy of rFSH and HMG could be a limitation of this analysis. Nevertheless, the oocytes yield per cycle could be considered a direct measure of gonadotropin stimulation and has been demonstrated to be highly correlated with live birth rate [10, 11]. Furthermore, Stoop et al. [35] demonstrated that a higher number of oocytes reduces cancelation rates, reduces the risk for multiple pregnancies and may lead to future pregnancies.

Any oocyte retrieved, independently from its maturation stage, was included in this analysis, due to the absence of information in the majority of the studies. However, Mehri et al. [36] showed that only mature oocytes have an increased fertilization rate.

The authors applied the Italian acquisition costs for HP-HMG to all HMG medications, whether highly purified or not. However, only a highly purified formulation is present in Italy. Furthermore, the cost of production related to a more sophisticated process could plausibly be more expensive, generating an underestimate of the costs associated with the HMG at a lower purification rate.

In conclusion, considering the number of oocytes retrieved as the best direct measure of efficient ovarian stimulation and considering the strong correlation between egg number and live birth, rFSH resulted to be more effective in comparison with HMG.

Despite a relatively high acquisition cost of rFSH, the use of recombinant therapy for the treatment of infertility in an Italian perspective generates a cost per oocyte similar to the cost associated to HMG due to higher oocytes yield.
